# Supplementary material for: Are carbon emissions trading and green financial instruments synergistic? -Comprehensive quantitative research based on content analysis
Source: PLoS One. 2024 Mar 7;19(3):e0298601. doi: 10.1371/journal.pone.0298601 (PMC10919601; doi:10.1371/journal.pone.0298601)
Supplement: S1 Data — (ZIP) [file pone.0298601.s001.zip › Data/地级市Z_F工作报告.zip!/─┌├╔╣┼/╬┌║ú╩╨/└┤╫╘╣1⁄2╓┌║┼í╛┬φ┐╦╩2╛▌═°í┐_2017.docx]

各位代表：

现在，我代表市人民政府，向大会报告政府工作，请予审议，并请政协委员和列席会议的同志们提出意见。

一、2016年工作回顾侯卫东官场笔记

过去的一年，是我市发展极为艰难、任务极为繁重的一年。面对错综复杂的外部环境和严峻的经济形势，我们始终坚定发展信心，坚决贯彻落实自治区党委、政府和市委的决策部署，全力推进稳增长、促改革、调结构、惠民生、防风险各项工作，经济社会保持平稳健康发展态势。全年地区生产总值完成572.2亿元，增长6.8%；一般公共预算收入完成81.56亿元，增长1.3%；固定资产投资完成165.12亿元，增长5.2%；社会消费品零售总额完成151.93亿元，增长9.3%；城镇和农区常住居民人均可支配收入分别达到36515元、15475元，均增长7.5%。

（一）全力以赴稳增长，经济运行企稳回升

把稳运行作为首要任务，出台了促进工业经济平稳增长若干政策意见，采取了一系列措施全力稳定企业生产。不断扩大电力多边交易范围，为51家企业减少电费支出4.1亿元。贯彻国家结构性减税政策，减免税收9.1亿元。设立过桥基金，帮助127家企业转续贷52.9亿元。充分发挥7支产业扶持基金作用，落实支持重点项目建设资金19.6亿元。创新中小微企业融资担保方式，为407户企业提供担保资金20.5亿元。企业融资成本持续下降，积极帮助企业协调银行贷款180亿元，全年贷款加权平均利率6.7%，同比下降1.1个百分点。大力支持非公经济发展，非公经济增加值完成398.8亿元，增长7%，非公经济对固定资产投资贡献率达52.3%。完善项目建设推进机制，开辟重点项目审批“绿色通道”，对符合行业准入、手续齐备的项目“即到即批”，18个自治区级重大项目开复工率达94%，10个自治区迎庆项目全部开复工。启动重点项目建设冬季行动计划，308个市级重点项目开复工196项，储备项目303个。

（二）坚定不移促转型，产业结构不断优化

工业产业转型步伐加快。工业增加值完成280.9亿元，增长5%。源通焦炉煤气制LNG项目投入试生产，华信焦炉煤气制LNG项目、家景镁业30万吨甲醇项目基本建成，“焦炭气化”从战略构想转变为现实生产力。神华乌海能源公司转型升级迈出可喜步伐。北京神雾科技40万吨聚乙烯多联产示范项目开工建设，引进航天十二院氢等离子体煤制乙炔技术，煤炭资源分级分质综合利用水平进一步提高。源宏医药中间体一期、亚东H酸和硫酸项目投入生产，煤焦化工新材料研究院特种炭黑项目开工建设，卡博特气相二氧化硅项目签约落地。天宇二期高岭土填料项目建成，鑫盛日电光伏项目实现并网发电，中钰镁合金轮毂及镁合金板型材项目开工建设，新能源、新材料、装备制造等战略性新兴产业占规模以上工业增加值的24%。乌海高新技术开发区被认定为自治区级高新技术产业开发区。

现代化农业稳步发展。第一产业增加值完成4.9亿元，增长3.7%。积极吸引社会资本，中广核光伏农业、华盛绿能光伏大棚等19个项目开工建设，总投资达25亿元。乌达区绿色生态产业园建成运营。成功举办中国•乌海2016“丝绸之路”世界沙漠葡萄酒文化节暨第22届全国葡萄学术研讨会，建立了中国乌海汉墨葡萄酒交易中心和国家质检总局葡萄酒区域检验检测中心，我市成为世界沙漠葡萄酒大赛永久举办地。加快农区土地流转，全年流转土地1.4万亩。推动农业龙头企业与农区居民建立利益联结机制，农户覆盖率达到85%。

现代服务业快速发展。第三产业增加值完成243.6亿元，增长9.9%。万达广场、月星家居投入运营，乌海北货场项目进展顺利，自治区西部首家跨境电商体验店开业，商贸、物流业占第三产业增加值比重达40%。游客服务中心投入使用，兰亭广场码头建成，新改建旅游厕所60个，22个乌海湖娱乐项目开业运营。乌海湖被评为国家水利风景区，当代中国书法艺术馆、蒙根花休闲农业园、阳光田宇国际酒庄被评为国家4A级旅游景区。桌子山召烧沟岩画被自治区认定为全区十个考古遗址公园之一。全年接待游客209.6万人次，增长13.4%；实现旅游收入40.6亿元，增长23%。设立全区首家“金融驿站”和“金融超市”，各类金融机构达到60家，银行存、贷款余额分别达到704.6亿元、789.7亿元，增长4.3%、7.6%。社会融资总额898.4亿元，直接融资占11.7%，达到历史最高水平。金融环境持续向好，成立金融仲裁院，金融机构贷款不良率1.8%，低于自治区平均水平。乌海银行发行绿色金融债券5亿元，我市成为中国西部首家发行绿色金融债券的地级市。金融业增加值占第三产业比重达到14.5%，金融业对税收的贡献度达到9.18%。

（三）建管并重提品质，城乡一体化水平稳步提高

规划引领作用充分发挥。启动城市总体规划和土地利用总体规划（2006—2020年）修编工作，开展近期建设规划（2016—2020年）编制及海勃湾老城区、滨河三期控规修改工作。编制完成环山湖旅游总体规划，以及城市综合管廊、交通运输、停车场等专项规划。

城市综合承载能力持续增强。交通网络不断优化，乌海湖大桥实现通车，石嘴山黄河大桥东引线、国道244线乌海段建成，市客运枢纽站主体基本完工，打通新海街、长青街等6条瓶颈路、断头路，完成19条街路、12.7万平方米路面改造，敷设道路排水管网32.5公里。旧城改造力度不断加大，完成房屋征收7910户，完成既有居住建筑节能改造50万平方米。改造热力管网31.5公里，新增供热面积124.7万平方米。城中水系建设步伐加快，整治河道5.1公里，新建人工湖12个。云计算中心投入使用，城乡4G网络覆盖率达到95%以上，我市被评为2016年度“宽带中国”示范城市。农区“十个全覆盖”工程全面完成，惠及农区群众6.3万人。

全国文明城市创建成效显著。举办“我的中国梦”等系列主题活动，文明新风日益形成。362个机关企事业单位开展包联共建，562个小区和平房区实现净化、亮化等 “五化”全覆盖。开展市容环境整治行动，新增垃圾箱1300个、停车场26处，城市面貌明显改观。市民服务中心主体建成，市民热线和数字城管成为国家级社会管理和公共服务综合标准化试点项目。

（四）深化改革增活力，对外开放水平不断提高

稳步推进供给侧结构性改革，积极落实“三去一降一补”五大任务，完成煤炭“去产能”30万吨，房地产“去库存”102.6万平方米。深入推进简政放权、放管结合、优化服务，编制公共服务事项目录，行政审批事项90%进驻市、区两级政务服务中心办理，实现“一窗”受理、“一站”审批、“一条龙”服务。加强事中事后监管，全力推进“双随机、一公开”监管改革，企业年报率达到90.6%。深化商事制度改革，启动“五证合一”工作，全年新注册企业1745家。积极推进不动产登记制度改革，在全区率先实现了统一登记发证。扎实推进信用体系建设，我市被列入国家第二批创建社会信用体系建设示范城市。医疗卫生体制改革成效显著，成立了以市人民医院、蒙中医院、妇幼保健院为核心的三个医疗联合体，实现了医疗资源向基层有序流动。全面完成党政机关和参公事业单位公务用车制度改革，节支率达7.4%。完成科技成果推广19项，获得专利授权116件，科技研发投入占GDP比重位居自治区前列。不断扩大开放，国际陆港、出入境国际旅行体检中心建成，乌海海关具备开关条件。我市与21个“一带一路”国家建立贸易往来，外贸进出口总额达8970万美元，增长93.9%。开展粤港澳招商活动，签约总额达52.9亿元。与意大利圣特拉姆市、法国利布尔纳市建立友好关系，在葡萄产业、体育、旅游、文化等方面开展务实合作。

（五）铁腕治理抓环保，生态文明建设成效明显

全面落实自治区关于乌海及周边地区大气污染联防联控工作部署，全力以赴抓好生态环境保护各项任务落实。全年投入社会治理资金56.14亿元，完成大气环境综合整治项目767个，占“十三五”任务总数的76.9%。扎实开展矿区环境综合整治，治理矸石自燃和煤层自燃840.4万平方米，修复整治排土场73个，硬化采区道路87公里。封闭物料堆场84.6万平方米，淘汰燃煤小锅炉190台，全面完成自治区下达任务。削减二氧化硫7600吨、氮氧化物1万吨，我市荣获自治区二氧化硫减排突出贡献奖。焦化企业污染防治走在全国前列。不断完善工业园区污水处理体系，工业污水基本实现达标排放。全力配合做好中央环保督察工作，建立市长包区、区长包园区、市区两级部门负责人包重点企业的三级包联责任制，我市成为中央环保督察期间群众举报环境污染案件最少的盟市之一。积极与鄂尔多斯市、阿拉善盟开展沟通协作，建立环境执法联动机制，区域大气污染联防联控成效明显。全年中心城区环境空气优良天数275天，达标率为75.1%，同比增加47天，空气质量显著改善。大力开展以“增绿、提质、增效”为重点的生态建设，组织实施了甘德尔山生态综合治理二期、矿区植被恢复试点、龙游湾湿地公园等50项生态建设工程，新建改造葡萄公园、书法广场等公共绿地19处，完成绿化面积531.7万平方米，建成区新增绿地面积20.6万平方米，绿地率达40.2%。我市首部地方性法规《乌海市海勃湾区生态涵养区保护条例》通过自治区人大常委会批准。全面落实最严格水资源管理制度，全国水生态文明城市建设试点任务基本完成。

（六）千方百计惠民生，社会保障水平稳步提升

坚持民生优先，全力推进社会保障、教育卫生、脱贫攻坚等十项重点民生工程，民生和社会事业投入同比增长12.4%，占一般公共预算支出的72.9%。

民生保障不断加强。积极推动创业带动就业，大众创业、万众创新取得实效，2家众创空间被认定为第三批国家级众创空间。扶持2339人成功创业，带动就业9177人。城镇登记失业率控制在自治区下达目标范围内。社会保障能力不断提升，城镇职工基本养老、基本医疗、失业、工伤、生育保险覆盖面进一步扩大。企业退休人员养老金提高至月人均2143元，增长7.7%。社会救助体系更加健全，最低生活保障标准居自治区前列。养老服务体系进一步完善，新建社区老年人日间照料中心和农区养老服务中心10个，建成老年公寓2个，市老年养护服务中心“公建民营”试点启动实施。全力推进保障性安居工程建设，全年新开工项目13个、9665套，开工率103.1%，居全区第2位；基本建成1.4万套，建成率174.2%。加强物业服务管理，全市住宅小区物业服务覆盖率达到86%，新交付小区物业覆盖率100%；加快“智慧小区”建设，签约小区106个。食品药品监管体系不断完善，完成食品药品检验检测资源整合和实验室升级改造。建成食品工业园2个，101家食品加工小作坊入驻运营。深入实施质量强市战略，君正化工等3家单位荣获首届市长质量奖。全力实施脱贫攻坚工程，制定精准帮扶措施，实行贫困户结对帮扶全覆盖，大力推进金融扶贫富民工程，全年减贫1424人。

社会事业全面进步。义务教育均衡发展，投入1.4亿元推进学校标准化建设，海勃湾区和海南区顺利通过国家义务教育发展基本均衡县评估验收。新改扩建幼儿园4所，学前三年毛入园率达到95%，居自治区前列。普通高考本科上线率达到76.7%，同比提高8.1个百分点。职业技术学院本科专业达到4个，乌海职业技能公共实训中心一期交付使用。医疗服务水平不断提升，市人民医院被评为全国第五家电子病历六级医院，介入技术达到国内先进水平；市妇幼保健院建成自治区西部首家生殖辅助中心，我市首例试管婴儿出生；市职业病防治院、精神卫生中心新院建成，蒙中医院二期主体完工。我市被确定为第二批国家医养结合试点。全面两孩政策稳妥有序实施。公共文化设施不断完善，桌子山召烧沟岩画遗址保护大厅展陈工程完工，群众艺术馆、文化馆全部进入国家等级行列，“书法五进”公共文化示范项目通过国家验收。成功承办第26届全国图书交易博览会，开展建市40周年大型美术主题创作等文化活动，举办“2016年乌海国际半程马拉松赛”等国内外品牌赛事活动13项，我市荣获“全国围棋之乡”称号，城市影响力进一步提升。

在做好各项工作的同时，我们不断加大自身建设力度，自觉接受人大和政协的监督，全年办结人大建议101件、政协提案134件，关于矿区环境污染治理的建议等33件建议提案列入市政府重点工作。深入开展“两学一做”学习教育，党员干部党性修养和能力素质进一步提升。强化行政监察和审计监督，推行财政预决算公开，市区两级预算决算和“三公”经费支出全部向社会公开。完善依法行政评议考核机制，健全纠错问责程序，梳理完善政府权责清单，普遍推行政府法律顾问制度，强化行政执法人员资格认定管理，政府工作法治化水平进一步提高。切实加强民主法制工作，推进“七五”普法。狠抓重点领域、重点环节部位的安全整治，开展安全生产大检查和专项检查32次，有效遏制重特大事故的发生。健全信访工作机制，社会矛盾纠纷大调解体系更加完善。社会治安综合治理不断加强，我市被国家确定为公共安全视频监控建设联网应用工程示范城市。我市被评为“全国无障碍建设示范市”、“全国双拥模范城”，连续七次被评为“自治区双拥模范城”。支持工会、共青团、妇联等人民团体开展工作，国防建设、民族宗教、档案史志、外事侨务、人防、气象、防震减灾、新闻出版、广播影视、无线电管理和红十字会等工作取得新成绩。

各位代表，过去的一年，面对宏观经济下行的严峻形势，广大干部凝心聚力、攻坚克难、扎实工作，特别是沙漠葡萄酒品牌创建、书法城建设、生态环境保护等工作得到自治区党委、政府的充分肯定和人民群众的普遍认可，各方面工作都取得了新进展、新成就。这些成绩的取得是自治区党委、政府和市委坚强领导的结果，是市人大、市政协鼎力支持的结果，是全市各族人民团结拼搏的结果。在此，我代表市人民政府，向全市广大干部群众，向所有关心、支持、参与乌海建设发展的社会各界人士，致以崇高的敬意和衷心的感谢！

在肯定成绩的同时，我们也要清醒地看到，经济社会发展中还存在一些困难和问题：一是经济运行不稳定、不确定因素较多，经济下行压力仍在延续，洗煤、焦化等企业恢复生产难度较大。二是固定资产投资乏力，项目储备不足，拉动经济持续增长的大项目、好项目较少。三是基础设施建设有待加强，城区统筹发展能力不强，旧城区基础设施建设和公共配套相对滞后；工业园区长期投入不足，基础设施成为制约工业经济持续发展的短板。四是服务业占GDP比重有所增加，但是总量和质量仍显不足。五是群众关心的空气质量有待进一步改善，环境保护和生态建设还需以更高的水平、更大的力度持续推进。同时，我们一些干部面对困难和压力存在不想为、不敢为、不愿为现象。对这些困难和问题，我们必须高度重视，进一步认真研究加以解决。二、2017年工作安排二号首长

2017年是实施“十三五”规划的重要一年和推进供给侧结构性改革的深化之年，我们将迎来党的十九大胜利召开和自治区成立70周年。尽管国内外经济形势依然严峻复杂，我市仍然面临着宏观经济下行和自身经济转型的双重压力，但我们更要看到有利条件和机遇。一是发展方向更加明确。2016年，李纪恒书记亲临乌海调研并作出重要指示，布小林主席先后四次调研乌海指导工作，都对我市今后一个时期的发展明确了定位、寄予了厚望，为我市加快发展提供了行动指南和根本遵循。二是发展信心更加坚定。自治区第十次党代会深刻把握时代脉搏、彰显内蒙古气魄，精心描绘了我区未来五年发展的宏伟蓝图，特别是首次明确提出“促进乌海及周边地区一体化发展”，为我市打造自治区西部区域中心指明了方向、提供了路径。我市第七次党代会立足乌海发展实际，旗帜鲜明地提出“五态一体”发展新思路，吹响了现代化乌海建设和转型发展的冲锋号角，必将鼓舞和激励全市人民砥砺前行，奋力夺取全面建成小康社会决胜阶段伟大胜利。三是发展动力更加强劲。经济转型成效明显，“焦炭气化”战略取得突破性进展，科技创新对产业转型升级引领作用更加突出，特别是全区2016年秋季重大项目集中开工建设动员大会召开之后，我市全面启动实施重点项目建设冬季行动计划，在全市上下掀起了抓项目、促投资的新热潮。2017年，随着乌海至宁东高速公路、包头至银川铁路客运专线、城市棚户区改造等一批重大基础设施和产业项目的开工建设，将持续拉动经济平稳增长。

做好2017年政府工作，必须全面贯彻党的十八大和十八届三中、四中、五中、六中全会精神，深入贯彻习近平总书记系列重要讲话精神和治国理政新理念新思想新战略，认真贯彻习近平总书记考察内蒙古重要讲话精神，全面落实中央经济工作会议、自治区第十次党代会和党委十届二次全会、市第七次党代会和市委七届二次全会决策部署，坚持稳中求进工作总基调，坚决守住三条底线，全力促进“五化”协同，深入推动“五态一体”发展，凝心聚力抓好投资和项目建设、经济转型和城市转型、产业结构调整、环境综合整治、棚户区改造、文明城市创建、区域协作发展等重点工作，统筹做好稳增长、促改革、调结构、惠民生、防风险、保安全等各项工作，全力谱写好祖国北疆亮丽风景线的乌海新篇章。

2017年发展的主要预期目标是：地区生产总值增长7.5%，一般公共预算收入增长-8.4%，固定资产投资增长16%，社会消费品零售总额增长9%，城镇常住居民人均可支配收入增长8%，农区常住居民人均可支配收入增长8.5%，节能减排完成自治区约束性控制指标，居民消费价格指数处于可控范围以内，城镇登记失业率控制在自治区下达目标以内。

围绕上述目标，重点抓好以下八个方面工作：

（一）坚守发展底线，以更大力度推进项目建设和投资

坚持发展第一要务，全力以赴稳定经济运行，把项目建设、投资和招商引资摆在经济工作的首要位置，保持经济中高速增长，决不滑出底线。

全力抓好项目建设。把2017年作为全市“重点项目建设年”，集中精力抓项目、扩投资、稳增长。全面实施重点项目建设冬季行动计划，争取开工一批、强力推进一批、全面建成一批、策划储备一批重大项目。全年计划实施重点项目303个，总投资1556亿元，其中10亿元以上项目37个。

全面强化分析调度。进一步落实领导干部包联重点项目责任制，形成一个项目、一名领导、一套班子、一抓到底的“四个一”推进机制。充分发挥综合经济部门联席会议作用，加强经济运行研判分析调度，实行“半月调度、集中研究，一企一策、精准帮扶”，切实帮助企业解决资金、用地、用水、用电等问题。支持非公有制经济和实体经济发展，加大对中小微企业扶持力度，完善政策措施，切实提振企业信心。继续扩大电力多边交易范围，启动输配电价改革试点，落实援企稳岗政策，进一步减免涉企税费，降低企业运营成本。推动建立焦化行业全产业链的价格联动机制，促成企业间风险共担、利益共享。推行工业用地弹性年期出让，对重点工业项目土地出让价款约定分期支付。

多措并举扩大投资。抢抓国家“一带一路”、乌海及周边地区一体化发展等战略机遇，重点围绕“七网”基础设施建设、棚户区改造、生态建设和环境保护等国家重点支持领域积极争取资金支持。制定出台招商引资优惠政策，发挥区位、土地、电价等综合优势，进一步增强招商引资实效。实施招商引资冬季行动计划，组建12支招商工作组，分赴京津冀、长三角、珠三角、川陕渝、环渤海、粤港澳等重点地区，积极引进产业转型升级、节能环保和产业转移项目。力争全年引进到位资金增长10%以上。进一步统筹乌海经济开发区“一区四园”发展，完善工业园区道路、管网、污水和危险废弃物处理等基础设施建设，积极申报乌海经济开发区为国家级经济技术开发区、低碳产业园为国家级高新技术产业开发区。制定出台“飞地经济”政策措施，积极对接发达地区产业转移项目，建立全市统一的产业准入、项目投资、权责对等、利益共享等方面政策体系，着力打造招商项目落地平台。鼓励民间资本进入教育、医疗、养老机构等社会事业和公用事业。继续完善PPP项目库，吸引社会资本投入城市基础设施建设。

（二）坚守生态底线，以更大决心抓好环境保护

环境保护是筑牢生态安全屏障的重要抓手，要提高到前所未有的高度来认识和推进，正确处理好经济发展同环境保护的关系，实现美丽与发展双赢。

坚决贯彻落实中央环保督察反馈意见，强化环境问题整改，重点实施煤田灭火工程和自然保护区内企业清理整顿工作，确保整改任务落实到位。突出抓好矿区环境综合整治，加快实施排土场和扬尘治理工程，扎实开展煤炭企业整顿和资源枯竭矿井关闭工作，完成矿区通道绿化、植被恢复治理等重点生态修复工程建设。加强大气污染防治，实施电力企业超低排放改造，严格控制扬尘等面源污染，淘汰矿区10蒸吨以下燃煤小锅炉，开展重点行业挥发性有机物治理。主动加强与鄂尔多斯市、阿拉善盟和石嘴山市等周边地区的沟通协调，协同推进区域大气污染联防联控。加大水污染防治力度，加强污水处理厂配套管网建设，提高企业中水回用比例。认真执行最严格水资源管理制度，强化水资源承载能力刚性约束。严格落实《乌海市海勃湾区生态涵养区保护条例》，进一步完善配套制度，确保饮用水安全。推行清洁生产，加大各类危险固废监管力度，突出抓好医疗废物深度处理和安全处置。严格落实耕地保护和节约集约用地制度，强化重点区域的土壤污染防治工作。加强环境执法监管，全面落实环境信用评价制度，加大对环境违法案件的查处力度，发现一起查处一起，决不姑息。

（三）坚守民生底线，以更普惠的政策增进人民福祉

坚持以人民为中心的发展思想，真情实意办好惠民利民实事，不断改善人民群众生活质量，让人民群众有更多获得感和幸福感。

坚决打赢脱贫攻坚战。深入实施精准扶贫、精准脱贫，持续落实“六个精准” 和“五个一批” 要求，制定精准产业帮扶措施，有效盘活现有土地、劳动力等资源，帮助解决启动资金、生产技术、产品销售等问题，增强贫困人口自我发展能力。实施金融扶贫工程，充分发挥国开行2.5亿元扶贫贷款作用。促进最低生活保障制度与扶贫开发政策有效衔接，将所有符合条件的贫困家庭纳入低保救助范围，做到应保尽保。加大医疗救助、临时救助、慈善救助等帮扶力度，防止因病致贫、因病返贫。建立动态管理机制，全力巩固提升脱贫成果。2017年基本完成脱贫攻坚任务。

切实保障和改善民生。全面落实就业创业扶持政策，深入推进大众创业、万众创新，提升创业园和创业孵化基地带动力。落实就业促进和创业引领计划，着力抓好以高校毕业生为主的重点群体就业创业工作。做好神华乌海能源公司等企业“去产能”职工安置工作，切实维护职工合法权益。年内城镇新增就业7500人以上，保持零就业家庭动态清零。完善社会保障体系，持续扩大社会保险参保范围，稳步推进“全民参保登记计划”和电子社保示范城市建设。完善基本医疗保险筹资和待遇调整机制，确保医保基金平稳运行。加大社会救助力度，适度提高最低生活保障和特困人员供养标准，保障困难群众基本生活。推进政府购买社区养老服务，鼓励和支持社会力量兴办社区老年人日间照料中心和养老服务中心。加大棚户区改造力度，实施下海勃湾二期、东山三期、海南拉僧仲等11个改造项目、1.2万户，切实改善棚户区居民的住房条件。

全面提升公共服务水平。高起点谋划学前教育发展，加快普惠性幼儿园建设进程，提高优质幼儿园比例，满足幼儿入园需求。推动义务教育优质均衡发展，确保乌达区通过国家义务教育发展基本均衡县评估验收。优化高中教育教学资源配置，推动普通高中优质特色多样化发展。加快发展中高等职业教育，进一步完善乌海职业技术学院基础设施，积极创建国家优质高职院校。充分发挥乌海职业技能公共实训中心作用，推动职业教育与产业发展紧密对接。高度重视民族教育和特殊教育发展。大力推进健康乌海建设，深化公立医院改革，推进分级诊疗制度建设。进一步完善蒙中医医疗服务体系，积极推进蒙中医药健康服务产业发展。成立民族医药产业基金，整合优化医疗资源，建设智慧医疗服务平台。深入开展爱国卫生运动，创建国家卫生城市。全面提升医疗服务水平，开工建设市人民医院新院，着力打造三甲医院。优化妇幼保健和计划生育服务，提高出生人口素质。启动生命纪念园改造工程，加快建设殡仪馆综合楼。推动现代公共文化服务标准化、均等化、专业化发展，抓好博物馆、市民文化活动中心新建工程。围绕书法文化建设，深入开展黄河明珠•中国乌海书法艺术节、“书法万里行”等大型节庆和文化交流活动，培育发展文博会展、书画奇石等特色文化产业，重点打造书法文化、甘德尔山蒙元文化、太阳神岩画文化、葡萄酒文化四大文化产业园区。广泛开展全民健身活动，努力打造10分钟健身圈，规划建设内蒙古西部足球训练基地。

（四）强力推进经济转型，在资源型城市转型上实现新突破

坚持创新驱动和项目拉动，以产业转型升级为主攻方向，改造提升传统产业，加快培育壮大战略性新兴产业，全力推动产业发展向中高端迈进。

创新驱动发展。树立绿色低碳循环发展理念，把创新作为转型升级的关键驱动，形成以创新为主要引领和支撑的经济体系和发展模式。加快煤焦生产加工基地向清洁能源生产加工基地转变。推进“焦炭气化”和煤气、煤焦油深加工，引导煤焦企业延伸发展LNG、甲醇、烯烃等下游产业链，推动源通、华信焦炉煤气制LNG、家景镁业30万吨甲醇等项目投产达效。加快氯碱化工向精细化、集约化转变。探索电石乙炔化工产业升级新路径，抓好北京神雾煤化科技40万吨聚乙烯多联产示范项目建设。深化煤焦化工、氯碱化工、硅化工融合发展，推动天津渤海精细化工促进剂、卡博特气相二氧化硅、北方食品甜味素等项目开工建设。加强内蒙古氯碱产品质量监督检验中心建设，促进氯碱产业提档升级。推进君正集团与上海华谊集团氯碱化工研究院合作，集中研发专用树脂和高性能树脂。加快战略性新兴产业向支柱性产业转变。培育壮大光伏产业，重点抓好采煤沉陷区光伏基地发电项目一期、巴音陶亥光伏农业小镇等项目。推动抽水蓄能电站建设，形成“多能互补”能源互联网新模式。全力争取将我市纳入“上海庙—山东”电力外送通道。夯实新材料产业发展基础，确保健翔碳纤维、无锡银阳新能源高纯硅等项目开工。加快中海外新能源汽车充电桩项目建设，推动中钰镁合金轮毂及镁合金板型材项目建成投产。积极创建国家第三批低碳城市试点。

军民融合发展。把军民融合发展战略作为推进转型发展的重要支撑，强化与航天十二院沟通对接，设立军民融合产业扶持发展基金，将氢等离子体煤制乙炔技术工业示范项目打造成为军民融合的龙头工程、精品工程。规划建设航天体验园，举办以经济转型升级为主题的“钱学森智库”高层论坛，着力打造军民融合创新平台。加强新型智慧城市、智慧园区技术合作，打造军民融合技术转移中心。

提升传统农业发展。加快推动传统农业向“生态、高效、特色、精品”的现代化农业转型，大力培育新型农牧业经营主体，培育壮大一批农业龙头企业、专业合作社和社会化服务组织。推动葡萄全产业链发展，加快汉森、阳光田宇、云飞、西口风等葡萄酒庄建设，全力打造“中国•乌海沙漠原生态葡萄酒庄之都”。深入推进农业供给侧结构性改革，把增加绿色优质葡萄供给放在突出位置，狠抓葡萄标准化生产、品牌创建、质量安全监管。建成国家级葡萄酒区域检测中心，推动中国乌海汉墨沙漠葡萄酒交易中心投入运营，强化与“一带一路”沿线国家葡萄酒文化交流，着力打造沙漠葡萄酒生产中心、文化中心。推动三次产业深度融合，大力推进集葡萄种植、贮运、加工及酒庄于一体的休闲农业发展，积极创建国家和自治区级休闲农牧业与乡村旅游示范点。扩大设施农业面积，不断提高蔬菜自给能力。加强粮食流通基础设施保障，推进第二粮库“粮安工程”建设。

（五）强力推进城市转型，在宜游宜居开放服务型城市建设上实现新突破

坚持规划先行，以规划引领城市转型发展，推动实施《乌海及周边地区城镇规划》，高标准做好《乌海市城市转型发展战略研究》。深化完善基础设施“七网”建设规划。加快编制《环乌海湖发展规划》。

打造宜游型城市。着眼发展全域旅游、四季旅游，实施“旅游+”战略，大力推动旅游与一二三产业融合发展，与“五化”协同发展，与产业建设、城乡建设、文化建设、民生建设、信息建设、生态建设一体发展，高标准抓好乌海湖及周边规划设计、开发建设。以资源组合、景区联合、线路整合、产业聚合为牵引，推动建立区域旅游合作机制和旅游服务网络，构建务实高效、互惠互利的乌海湖及周边地区旅游合作体。发展特色旅游商品、旅游装备制造、自驾游服务，打造中国西部旅游集散中心和国际特色旅游目的地。力争全年接待游客达到240万人次以上、旅游收入突破50亿元。做好“水”的文章。按照“七网+旅游”的思路，加快完善旅游基础设施网络。完善环乌海湖旅游码头、绿道、停车场、旅游厕所等基础设施，加快乌海院子城市休闲度假区等项目建设，办好“丝绸之路”中国•乌海沙漠葡萄酒文化节、乌海湖沙漠越野、马拉松、环湖自行车等重大节庆赛事，打造集观光旅游、体育竞技、文化活动于一体的乌海湖黄金岸线。多渠道、全方位宣传乌海湖旅游，积极创建乌海湖国家级旅游度假区，让乌海湖扬名天下。做好“山”的文章。加快甘德尔山生态文明景区游客中心、玻璃廊桥等设施建设，全力打造蒙元文化、草原文化、赏石文化等特色景区。完成桌子山召烧沟岩画博物馆附属工程。加快高山草甸项目建设。依托满巴拉僧庙，举办内蒙古传统蒙医药文化旅游节，打造区域蒙医蒙药产业中心。做好“沙”的文章。高标准、高起点整合沙漠、湖区沙岛、金沙湾等资源，培育沙疗养生、沙漠徒步、沙漠越野等沙漠休闲运动项目，逐步形成以商务会展、健康养生、沙滩运动为特色的沙漠休闲度假基地。

打造宜居型城市。继续推进“六城联创”，动员全社会各方面力量，大力开展文明礼仪宣传等主题活动，突出抓好交通秩序管控、环境卫生清洁等工作，创建成为全国文明城市。加强城市基础设施建设，完善城区道路网络，新建改造光明路、东环路等49条街路，打通城区断头路、瓶颈路，改造街路26.3万平方米。加快完善旧城基础设施，逐步将旱厕改造成水冲式新型公厕。积极申报国家综合管廊试点城市，开工建设海勃湾区建设路6公里地下综合管廊。完善城市服务功能，新建热力站5座、一次支线管网6公里，供热普及率达到90%。积极推进海勃湾区150公里灰口铸铁燃气管网改造，供气普及率达到94%。加快海勃湾区北部净水厂、乌达区净水厂、海南区净水厂建设，建立全市统一的水质检测中心，保障居民饮水安全。大力推进环卫工作市场化改革，扩大城乡垃圾收集覆盖面，机械化清扫率达到80%。推动餐厨垃圾资源化利用和无害化处理项目投入运营，完成医疗废物集中处置中心二期新建工程，生活垃圾无害化处理率达到98.5%。提升城市绿色生态品质，大力推进京津风沙源治理、天然林保护等国家重点林业工程，加快乌海湖右岸、甘德尔河岸等绿化项目建设。实施城中、城北、绕城“三大水系”工程，打造集城市景观、文化旅游和防洪功能于一体的城市循环水系，力争完成全国水生态文明城市建设。

打造开放型城市。按照自治区推进乌海及周边地区一体化发展战略，推动自治区出台实施意见，制定专项规划，进一步明确区域协作目标、路径和举措。积极推动乌海及周边地区转型升级，创新区域合作模式，推进区域基础设施一体化、园区布局一体化、公共服务一体化，实现要素整合、资源共享、优势互补，切实改善区域环境质量。积极加强与周边地区交流合作，主动推进区域协同发展。加强与呼包银榆经济区各城市的沟通联系，承办2017年呼包银榆经济区联席会议。积极扩大对外开放，加强与粤港合作、向北开放，主动融入国家“一带一路”发展战略，把握乌力吉口岸开放有利契机，大力发展对外贸易。深化海关大通关改革，全面推行“三个一”联合监管模式。与意大利圣特拉姆市建立友好城市关系，积极与蒙古国达尔汗乌拉省、法国利布尔纳市进行互访，深化工业、葡萄产业、旅游体育、医疗等方面的务实合作。与自治区出入境检验检疫局共同建成检验检测科学院，努力打造自治区西部检验检测中心。

打造服务型城市。进一步完善服务业发展政策措施，成立专门机构对全市服务业发展进行统一规划管理、协调推进，整合完善“七网”基础设施，推动产城融合，提高城市综合服务水平。建设自治区西部区域重要的交通中心。完善乌海机场基础设施建设，优化航线网络布局。配合做好包银高铁乌海段前期工作，力争早日开工建设。争取S37线乌海至宁东高速公路等重大项目开工建设，推进国道110线乌海黄河特大桥建设，完成荣乌高速乌海段工程。建设自治区西部区域物流中心。加快乌海北货场等项目建设，推动乌海国际陆港投入使用、乌海海关通关运行，争取设立乌海保税仓，使我市成为“丝绸之路经济带”上连接欧亚大通道、辐射蒙晋陕甘新和蒙古的重要物流枢纽。建设自治区西部区域金融中心。组建完成金融控股集团，进一步完善金融体系。制定区域金融服务中心发展规划，推动中信银行设立分支机构、君正集团设立财务公司，支持乌海银行增资扩股。设立“助保贷”基金，积极为中小企业融资增信。组建各类产业发展基金，重点支持产业转型升级和新兴产业项目建设。完善多层次资本市场体系，培育上市企业，在新三板挂牌有所突破。建立政银企联动机制，积极化解银行不良贷款，确保不发生区域性、系统性金融风险。建设自治区西部区域信息中心。借助自治区打造国家大数据综合试验区的机遇，强化与清华同方、浪潮、华为、腾讯等信息技术产业龙头企业的合作对接，依托云计算中心，加快大数据中心和大数据产业园建设。编制电子商务发展规划，出台促进电子商务发展的实施意见，建设乌海信用信息体系，加快实施“互联网+”10大行动计划。完善光纤宽带网络、免费WiFi和物联网等信息基础设施，建设智慧公共交通运输服务平台、智慧消防物联网系统。建设自治区西部区域人才集聚中心。深化人才发展体制机制改革，大力实施人才强市战略和“草原英才”工程，深入推进人才工作“五项计划” 。加强各类科研机构、创客空间等平台建设，集聚一批产业领军人才和人才团队。推进产学研结合、校企合作，大力培养技能人才。完善人才服务体系，充分发挥人才开发专项资金作用，完善教育、医疗、养老、住房保障等配套措施，促进各类人才扎根乌海、奉献乌海。

（六）突出抓好重点领域改革，充分释放社会活力

贯彻落实好国家、自治区和市委全面深化改革的部署要求，深入推进供给侧改革，认真抓好“三去一降一补”五大任务落实，重点围绕振兴实体经济、促进房地产市场平稳健康发展等方面推出一批重大改革举措。大力推进简政放权、放管结合、优化服务，简化行政审批和公共服务事项流程，清理规范中介服务事项，推进“一号一窗一网” 服务模式改革。全面推行“双随机、一公开” 监管，营造阳光公平的市场环境。深化投融资体制改革，优化投资结构，增加有效投资。创新政府与社会资本合作模式，吸引民间投资重点投向产业转型项目和基础设施建设。深化财税体制改革，制定财政资金支出审批管理办法，改进预算管理制度，建立全面规范、公开透明的现代预算制度。完善促进非公经济发展的机制和政策，打造良好营商环境，构建“亲”“清”新型政商关系。推进户籍制度改革，鼓励农区居民进城购房，消化房地产库存。深化农区产权制度改革，推进农区土地所有权、承包权、经营权“三权”分置并行。做好海勃湾、乌达农区土地确权登记颁证工作，推动土地有序流转。积极稳妥推进事业单位、国有企业公务用车制度改革。

（七）突出抓好社会治理，切实维护社会和谐稳定

树立安全发展理念，把2017年作为全市“安全生产年”，推动安全生产工作常态化开展。严格落实安全生产责任制，坚持“党政同责、一岗双责、齐抓共管、失职追责”，强化部门监管责任、企业主体责任落实。深入开展安全生产大检查，加强重点领域专项整治，坚决防范遏制重特大事故发生。构建风险分级管控和隐患排查治理双重预防机制，提高安全生产专业化、信息化水平，切实保障人民生命财产安全。

健全市、区、街道（镇）、社区网络化管理，加快推进以“智慧小区”为核心的“互联网+”社区管理。完善物业管理机制，规范物业企业服务行为，提高物业管理水平。落实重大决策社会风险评估制度，推进社会矛盾多元化解，着力打造阳光信访、责任信访、法治信访，维护群众合法权益。进一步加强食品药品安全监管，建成食品药品安全信息化监管平台，启动追溯体系建设，建立完善食品安全检验检测体系，打造食品药品安全城市。严厉打击各类违法犯罪活动，构建立体化社会治安防控体系，努力建设更高水平的平安乌海。做好自治区成立70周年迎庆工作。充分发挥工会、共青团、妇联等人民团体作用，关心支持妇女儿童、残疾人、慈善救助等事业，继续做好民族宗教、国防建设、双拥优抚、人防、气象、地震、档案史志、新闻出版、广播影视、无线电管理等各项工作。

（八）更加注重自身建设，建设务实勤政法治廉洁政府

新形势新任务对政府工作提出了新要求。我们将切实增强“四个意识”特别是核心意识、看齐意识，始终与以习近平同志为核心的党中央保持高度一致，做到明责担责尽责，用心用情用力做好各项工作，着力打造忠诚干净担当的高素质干部队伍。

建设务实政府。始终坚持不折不扣地贯彻落实中央、自治区和市委各项安排部署，始终保持重大决策部署的连续性、稳定性和创新性，始终依靠全市四大班子的密切协作和广大干部群众的团结奋斗，做到一张蓝图绘到底、一以贯之抓落实。立足乌海转型发展大局，不贪一时之功，不图一时之名，多做打基础、利长远的好事实事要紧事。

建设勤政政府。大力发扬“蒙古马”和钉钉子精神，树立“事不过夜、只争朝夕”的工作理念，保持与时俱进、锐意改革、开拓创新的精神风貌。坚持积极行政、主动作为，不为困难找理由，只为成功找办法。抓住想干事、敢干事这两个关键点，健全干部正向激励机制，为敢于担当的干部担当，为敢于负责的干部负责。进一步解放思想，跳出思维定势，创新工作方法，形成敢作敢为、善作善成的良好氛围。

建设法治政府。深入推进依法行政，严格按照法定权限、法定程序履职尽责，把政府全部工作纳入法治轨道。依法接受人大法律监督、工作监督和政协民主监督，高度重视民主党派和无党派人士提出的意见、批评、建议。完善政府重大决策合法性审查机制和法律顾问制度，健全重大问题专家咨询、听证等制度。推进“七五”普法，做到尊法学法守法用法。

建设廉洁政府。坚持把纪律和规矩挺在前面，严格执行《关于新形势下党内政治生活的若干准则》和《中国共产党党内监督条例》。充分发挥政府党组核心领导作用，以“两学一做”学习教育为重要载体，持续加强作风建设，确保全面从严治党主体责任落地生根。严格落实党风廉政建设“一岗双责”，贯彻执行廉洁自律准则、问责条例和纪律处分条例。完善权力运行制约和监督机制，强化土地出让、产权交易、政府采购等重点领域、关键环节监管。加强行政监察和审计监督，推进政务信息公开，确保权力在阳光下运行。

各位代表，让我们更加紧密地团结在以习近平同志为核心的党中央周围，在自治区党委、政府和市委的坚强领导下，守望相助、团结奋斗、一往无前，为开创乌海转型发展新局面，谱写全面建成小康社会新篇章，打造祖国北疆亮丽风景线作出新的、更大的贡献而努力奋斗！
